# Neuropsychiatric systemic lupus erythematosus subtypes identified by unsupervised clustering: A single-center cohort study

**DOI:** 10.1515/rir-2026-0002

**Published:** 2026-04-08

**Authors:** Weiting Fang, Jiuliang Zhao, Dong Xu, Ziqian Wang, Mengtao Li, Siyuan Fan, Tao Li, Manqing Xie, Zeyu Liu, Hui You, Yanhong Wang, Shangzhu Zhang, Xiaofeng Zeng

**Affiliations:** Department of Rheumatology and Clinical Immunology, Peking Union Medical College Hospital, Chinese Academy of Medical Sciences & Peking Union Medical College, Beijing, China; National Clinical Research Center for Dermatologic and Immunologic Diseases (NCRC-DID), Ministry of Science & Technology, Beijing, China; State Key Laboratory of Complex Severe and Rare Diseases, Peking Union Medical College Hospital, Beijing, China; Key Laboratory of Rheumatology and Clinical Immunology, Ministry of Education, Beijing, China; Department of Neurology, Chinese Academy of Medical Sciences & Peking Union Medical College, Beijing, China; Department of Psychology, Chinese Academy of Medical Sciences & Peking Union Medical College, Beijing, China; Department of Radiology, Chinese Academy of Medical Sciences & Peking Union Medical College, Beijing, China; Department of Epidemiology and Biostatistics, Institute of Basic Medical Sciences Chinese Academy of Medical Sciences & School of Basic Medicine Peking Union Medical College, Beijing, China

**Keywords:** neuropsychiatric systemic lupus erythematosus, autoantibodies, cluster

## Abstract

**Background and Objectives:**

Neuropsychiatric (NP) manifestations are common, heterogeneous and severe in systemic lupus erythematosus (SLE) patients, with attribution to SLE remaining diagnostically challenging. Traditional classification focuses on clinical syndromes, overlooking neuropsychiatric systemic lupus erythematosus (NPSLE) immunological heterogeneity. To address the heterogeneity of NPSLE, this study aimed to delineate distinct disease subgroups by clustering patients based on their antibody profiles. These subgroups were then evaluated for diferences in clinical presentation and prognosis, to better characterize disease subsets and support individualized approaches to diagnosis and management.

**Methods:**

This retrospective single-center study included hospitalized SLE patients with NP manifestations, collecting demographic, clinical and laboratory data. Patients were classified as NPSLE or non-NPSLE by clinical judgment after excluding alternative causes. Hierarchical cluster analysis explored autoantibody-clinical feature associations.

**Results:**

Among the 167 patients analyzed, 152 had NP manifestations attributed to SLE. Central nervous system (CNS) involvement was predominant (89.1%), with seizures, cerebrovascular disease, acute confusional state (ACS), and demyelinating syndrome being most prevalent manifestations. Hierarchical clustering of 152 NPSLE patients identified two subgroups: Cluster 1 (23.7%) demonstrated cerebrovascular injury as the predominant manifestation, with higher positivity rates of antiphospholipid antibodies (APLs) (*P* < 0.01) and a higher incidence of cerebrovascular disease (*P* < 0.01). Cluster 2 (76.3%) showed immune-mediated inflammatory profile, with higher positivity of anti-SSA (*P* < 0.01), antidsDNA (*P* < 0.05), and anti-RiboP antibodies (*P* < 0.05). Neurological involvement predominantly manifesting as ACS (*P* < 0.05), accompanied by a higher frequency of fever and joint involvement.

**Conclusions:**

In this study, NPSLE exhibited distinct serological profiles and segregated into two immunologically defined clusters, reflecting its clinical and biological heterogeneity, and suggesting that immunological profiling may enhance precise classification and personalized management of affected patients.

## Introduction

Systemic lupus erythematosus (SLE) is a chronic autoimmune disease affecting multiple systems.^[1]^ Neuropsychiatric systemic lupus erythematosus (NPSLE) is one of its most severe complications of SLE, with reported prevalence of nervous system involvement varying widely, ranging from 4% to 91%.^[2,3]^ The pathogenesis of NPSLE remains incompletely understood, but it is thought to primarily involve two principal mechanisms: ischemic and inflammatory. These mechanisms may overlap and act concurrently, contributing to the marked clinical heterogeneity of the disease. The ischemic pathway is often associated with focal neuropsychiatric (NP) manifestations such as stroke, seizures, and movement disorders. It is mediated by antiphospholipid antibodies (APLs), immune complexes, and complement activation, potentially affecting intracranial blood vessels of various calibers.^[4, 5, 6]^ In contrast, the inflammatory pathway is more closely linked to diffuse manifestations like psychosis and acute confusional state (ACS).^[7]^ Key mechanisms underlying this pathway include increased blood-brain barrier permeability, microglial activation, synaptic pruning, and intrathecal immune complex formation, which triggers plasmacytoid dendritic cell activation and the production of type I interferons and other pro-inflammatory cytokines and mediators.^[2]^

NP manifestations in NPSLE encompass a broad spectrum of clinical features. According to the 1999 American College of Rheumatology (ACR) classification criteria for NPSLE, these manifestations are categorized into 19 distinct NP syndromes.^[8]^ Notably, SLE can affect the central nervous system (CNS), peripheral nervous system (PNS), and autonomic nervous system, resulting in neurological symptoms that range from mild cognitive impairment to seizures and cerebrovascular events. Furthermore, based on anatomical distribution and clinical severity, CNS manifestations are commonly classified as either focal or diffuse syndromes and further categorized as minor or major events based on their clinical impact.^[8, 9, 10]^

Although NPSLE presents with a wide range of clinical manifestations, none are specific to the condition. Currently, no single laboratory or imaging biomarker can accurately diagnose NPSLE.^[11]^ In clinical practice, diagnosis often relies on a physician expertise, supported by neuropsychological testing, cerebrospinal fluid analysis and neuroimaging. These objective assessments may strengthen diagnostic confidence but do not provide definitive confirmation. The overlap between NPSLE symptoms and those of other neuropsychiatric disorders with different etiologies further complicates differential diagnosis. To address this challenge, various attribution models for NP symptoms have been developed to help identify primary NPSLE. One such model is the Italian model proposed by Bortoluzzi *et al*. in 2015.^[12]^ However, it is essential to note that these models serve only as diagnostic aids and should not be considered fully reliable for attributing NP symptoms.^[13]^ Ultimately, comprehensive clinical evaluation remains the primary standard for diagnosing NPSLE.

Despite increasing recognition of NPSLE as a severe complication of SLE, its clinical and immunological heterogeneity remains incompletely defined. Few studies have systematically characterized the spectrum of clinical phenotypes and associated autoantibody patterns in patients with NPSLE. To address this gap, we conducted a cluster analysis based on autoantibody expression profiles to identify distinct subgroups of NPSLE. By elucidating potential immunological signatures and their associations with clinical manifestations and disease outcomes, our study provides new insights that may inform individualized diagnosis and inform future mechanistic investigations.

## Patients and Methods

### Patient Selection

This study enrolled patients hospitalized in the Department of Rheumatology and Immunology at Peking Union Medical College Hospital (PUMCH) between 2015 and 2019. All patients fulfilled either the 1997 ACR classification criteria ^[14]^ or the 2012 Systemic Lupus International Collaborating Clinics classification criteria for SLE.^[15]^ The diagnosis of NPSLE was based on neurologic and psychiatric syndromes involving the nervous system as classified by the ACR Subcommittee in 1999.^[16]^ According to the ACR case definitions, headaches were classified using the International Headache Society criteria,^[17]^ while mood disorders are diagnosed based on clinical judgment following the Diagnostic and Statistical Manual of Mental Disorder, Fourth Edition (DSM-IV).^[18]^ To ensure that neuropsychiatric events could be attributed to NPSLE, all patients in this study were hospitalized and underwent a comprehensive, systematic evaluation by a team of experienced medical professionals during their stay to minimize confounding factors that could affect diagnostic accuracy. Medical records were subsequently reviewed in detail, and cases with missing key information were excluded. For patients with ambiguous documentation, clinical data were collected and reviewed by a multidisciplinary team (MDT) comprising two rheumatologists, one radiologist, one psychologist and one neurologist. In addition, patients who had confirmed infections or other identified etiologies for NP symptoms prior to hospitalization were excluded from the study. All patients received long-term immunosuppressive therapy following discharge and were routinely monitored through outpatient follow-up. When routine follow-up records were unavailable, telephone follow-ups were conducted to obtain information regarding neuropsychiatric status. Recurrence is defined as the first NPSLE episode requiring hospitalization after enrollment.^[19]^ As this was a retrospective study based on medical records obtained for clinical purposes, the requirement for informed consent was waived, and confidentiality protocols were followed.

### Data Collection

Comprehensive clinical data were collected, including demographic characteristics, clinical manifestations, laboratory findings and Systemic Lupus Erythematosus Disease Activity Index 2000 (SLEDAI-2K) scores. Follow-up status and clinical outcomes were also recorded, with NPSLE recurrence defined as the primary endpoint event during follow-up.

### Statistical Analyses

Categorical data are summarized as frequencies and percentages, while continuous variables are expressed as medians with interquartile ranges (IQRs). The Chi-square test and Fisher’s exact test were applied for categorical variables. Statistical significance was set at *P* < 0.05. Prior to cluster analysis, the completeness of the 11 autoantibodies (anti-ds-DNA, anti-RiboP, anti-Sm, anti-SSA, anti-SSB, anti-Ro52, anti-SCL70, anti-RNP, lupus anticoagulant [LA], anti-cardiolipin antibodies [aCL], anti-β2-glycoprotein I) was systematically verified. All included patients were hospitalized and underwent comprehensive serological testing during admission. The overall missing rate across autoantibody variables was low (2.3%) and showed no evidence of a selective missing pattern. Given the low missing rate (< 5%), a complete case analysis was performed.

Cluster analysis was conducted based on the antibody expression profiles of patients with NPSLE, and the resulting subgroups were subsequently compared with respect to clinical features and prognostic outcomes. Prior to analysis, all variables were converted into binary (positive/negative) categories. Hierarchical clustering was performed on all binary variables using the Hamming distance and average linkage method. The “NbClust” package of R software was used to determine the optimal number of clusters.^[20]^ The cumulative incidence function (CIF) was applied to estimate the cumulative incidence of disease recurrence between the two clusters, accounting for competing events (*e.g*., death). Differences between clusters were tested using Gray’ s test. Cumulative incidence curves were generated with the “tidycmprsk” package and visualized with the “ggplot2” package. All statistical analyses were performed using R software (v3.6.1; R Foundation for Statistical Computing, Vienna, Austria).

## Results

### Clinical Characteristics of Patients

**Figure 1:**
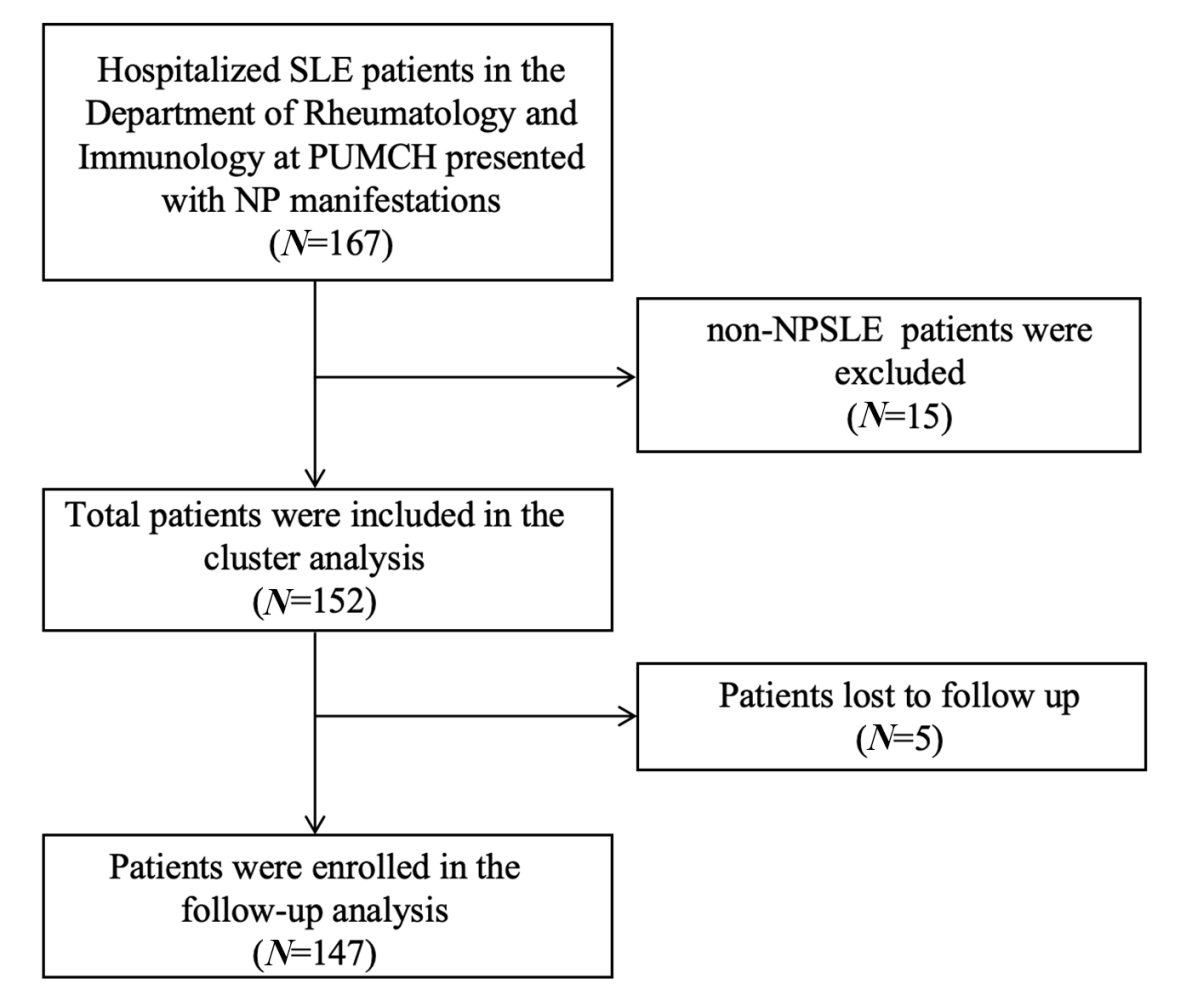
Study flowchart. non-NPSLE: neuropsychiatric manifestations not attributed to SLE; PUMCH: Peking Union Medical College Hospital; SLE: systemic lupus erythematosus.

A total of 167 patients with NPSLE patients were enrolled, and their medical records were systematically reviewed. After excluding 15 patients with secondary non-NPSLE etiologies—including 8 with infections, 2 with drug-induced toxicity, 1 with posterior reversible encephalopathy syndrome, 1 with hematologic disorders, 1 with metabolic disturbances, 1 with autoimmune encephalitis and 1 with postpartum depression—152 patients were ultimately classified as having SLE-associated neuropsychiatric involvement and were included in the analysis (Figure 1). The cohort had a median age of 30 years (IQR 25–36) and a median disease duration of 3 years (IQR 0.5–7.0), defined as the time interval from the initial diagnosis of SLE to hospital admission. At baseline, APS was identified in 24 cases (15.8%) and renal involvement was present in 95 patients (62.5%). Serological profiling demonstrated anti-SSA antibody positivity in 85 (55.9%) of cases, anti-Ribop antibody positivity in 38 (25%) of cases, as well the presence of lupus anticoagulant 50 (32.9%), anti-β2 glycoprotein 1 antibodies 37 (24.3%) and anticardiolipin antibodies 39 (25.7%). Complement C3 levels were reduced (median 0.6 g/L, IQR 0.4–0.8) (Table 1). Pulse glucocorticoid therapy (intravenous drip of methylprednisolone ≥ 500 mg daily for three consecutive days) was administered to 94 patients (61.8%), which continued with oral prednisone (1 mg/kg/day) for 4 weeks and subsequent tapering. All other patients received adequate-dose glucocorticoids (1 mg/ [kg·d] prednisone or an equivalent dose of other glucocorticoids, administered continuously for ≥ 2 weeks). Immunosuppressive therapy was administered to 148 patients (97.4%); of these, 107 (70.4%) received cyclophosphamide (CTX) alone, 33 (21.7%) received mycophenolate mofetil (MMF) alone (Table 1).

**Table 1:** Baseline demographic and clinical characteristics of the study population

Characteristics	NPSLE (*n* = 152)
Age (years, median [IQR])	30 [24, 38]
Sex, male, *n* (%)	15 (9.9)
Duration of SLE (years, median [IQR])	3 [0.5, 7.0]
Combined Antiphospholipid syndrome, *n* (%)	24 (15.8)
SLEDAI-2K (median [IQR])	13 [8–22]
Systemic involvement of SLE, *n* (%)	
ACLE/SCLE	47 (30.9)
CCLE	4 (2.7)
Alopecia	47 (30.9)
Photoallergic	18 (11.8)
Mucosal ulcers	32 (21.1)
Muscle involvement	3 (2.0)
Joint involvement	61 (40.1)
Renal involvement	95 (62.5)
Thrombocytopenia	63 (41.4)
Leukopenia	57 (37.5)
Hemolytic anemia	12 (7.9)
Myocardial damage	11 (7.2)
Interstitial lung disease	8 (5.3)
Pulmonary arterial hypertension	9 (5.9)
Fever	63 (41.4)
Serological features at enrollment	
Positive Antibodies, *n* (%)	
Anti-Sm	25 (16.4)
Anti-dsDNA	83 (54.6)
Anti-SSA	85 (55.9)
Anti-SSB	22 (14.5)
Anti-Ro52	73 (48.0)
Anti-RiboP	38 (25.0)
Anti-RNP	49 (32.2)
Anti-Scl-70	1 (0.7)
LA	50 (32.9)
Anti-β2GP1 antibody	37 (24.3)
aCL	39 (25.7)
Positive Coombs test, *n* (%)	68 (44.7)
ESR (mm/h, median [IQR])	23[10.0, 50.0]
hsCRP (mg/L, median [IQR])	2.6 [0.6, 7.8]
C3 (g/L, median [IQR])	0.6 [0.4, 0.8]
C4 (g/L, median [IQR])	0.1 [0.05, 0.14]
IgG (g/L, median [IQR])	13.3[10.5, 18.0]
Treatment	
GC dose, *n* (%)	
pulse GCs	94(61.8)
1 mg/kg/day	58(38.2)
CTX, *n* (%)	107(70.4)
MMF, *n* (%)	33(21.7)

aCL, anticardiolipin antibody; ACLE, acute cutaneous lupus erythematosus; β2GP1, β2-glycoprotein-1; CCLE, chronic cutaneous lupus erythematosus; C3/C4, complement 3/4; CTX, cyclophosphamide; dsDNA, double-stranded deoxyribonucleic acid; ESR, erythrocyte sedimentation rate; GC, glucocorticoid; hsCRP, high-sensitivity C-reactive protein; IgG, immunoglobulin G; LA, lupus anticoagulant; MMF, mycophenolate mofetil; NPSLE, neuropsychiatric systemic lupus erythematosus; RNP, ribonucleoprotein; RiboP, ribosomal P protein; SLE, systemic lupus erythematosus; SCLE, sub-acute cutaneous lupus erythematosus; Sm, Smith; SCL-70, topoisomerase I; SSA, Sjogren’s syndrome antigen A; SSB, Sjogren’s syndrome antigen B; SLEDAI-2K, Systemic lupus erythematosus disease activity index 2000.

### Neurological Manifestations and Laboratory Characteristics of Patients with NPSLE

**Figure 2:**
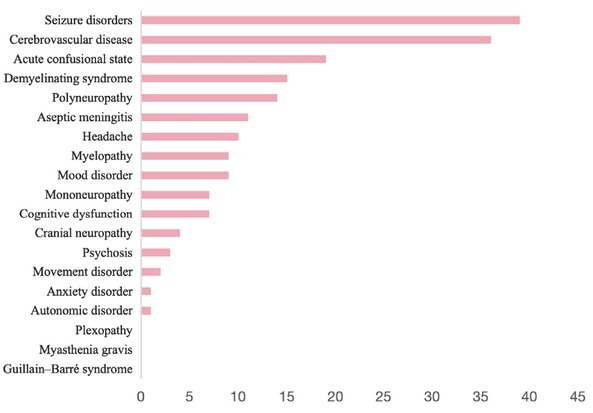
Distribution of subtypes of neuropsychiatric manifestations in NPSLE. NPSLE: neuropsychiatric systemic lupus erythematosus.

Among patients with NPSLE, 16 NP subtypes were identified. In total, 152 patients experienced 187 neuropsychiatric manifestations. Of these, 127 (83.5%) had isolated CNS involvement, 17 (11.2%) had isolated PNS involvement, and 8 (5.3%) had combined CNS and PNS involvement (Supplementary Table S1). Within the CNS, there were 161 NP manifestations, comprising 49 diffuse and 112 focal manifestations (Supplementary Figure S1). The most common NP manifestations included seizures, cerebrovascular disease (34 ischemic events, 2 hemorrhagic events), ACS and demyelinating syndrome (Figure 2, Supplementary Table S2). Among 139 patients who underwent lumbar puncture, increased cerebrospinal fluid (CSF) pressure was observed in 46 cases (33.1%), while elevated CSF protein levels were detected in 53 patients (38.1%) (Supplementary Table S3).

### Cluster Analysis

Cluster analysis was performed using autoantibody detection data from 152 patients with NPSLE. The variables included: anti-dsDNA, anti-RiboP, anti-Sm, anti-SSA, anti-SSB, anti-Ro 52, anti-SCL 70, anti-RNP, LA, aCL and anti-β2-glycoprotein 1 (β2GP1) antibodies. Using the Calinski–Harabasz (CH) index, the optimal number of clusters was determined to be two (Supplementary Figure S2).

**Figure 3:**
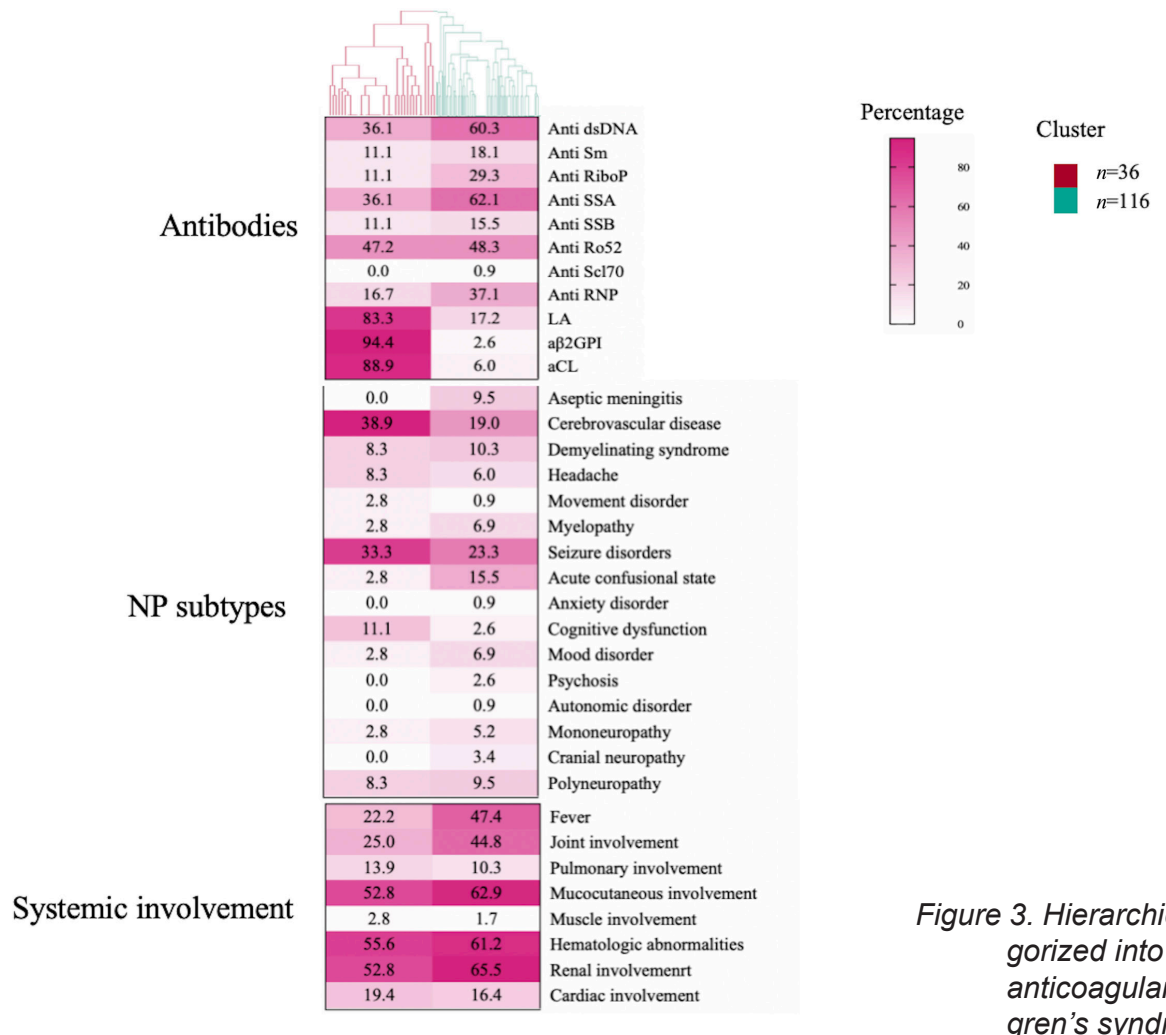
Hierarchical clustering analysis of 152 patients with SLE, categorized into two clusters. aCL: anticardiolipin antibody; LA: lupus anticoagulant; SSA: Sjogren’s syndrome antigen A; SSB: Sjo-gren’s syndrome antigen B; SLE: systemic lupus erythematosus.

Based on this result, two distinct clusters were identified. We then examined differences in NP symptoms and organ involvement between patients in each cluster (Figure 3). Cluster 1 (23.7%) manifested predominant cerebrovascular ischemic and hemorrhagic injury, with significantly higher antiphospholipid antibody positivity (*P* < 0.001) and a higher prevalence of cerebrovascular disease (38.9%, *P* < 0.01). Cluster 2 (76.3%) displayed systemic immune-mediated inflammatory profile, with significantly higher positivity of anti-SSA (*P* < 0.01), anti-dsDNA (*P* < 0.05), anti-RNP and anti-RiboP antibodies positivity (*P* < 0.05). Clinically, this cluster predominantly presented with ACS (15.5%, *P* < 0.05) along with significantly higher frequencies of fever (47.4%, *P* < 0.01) and joint involvement (44.8%, *P* < 0.05) (Table 2).

**Table 2:** Comparison of NPSLE subtype and antibody profiles between clusters

Characteristics	Cluster 1 (*n* = 36)	Cluster 2 (*n* = 116)	*P* value
NPSLE subtype			
Cerebrovascular disease	14 (38.9)	22 (19.0)	**0.024**
Seizure disorders	12 (33.3)	27 (23.3)	0.227
Aseptic meningitis	0 (0)	11 (9.5)	0.067
Polyneuropathy	3 (8.3)	11 (9.5)	1.000
Demyelinating syndrome	3 (8.3)	12 (10.3)	1.000
Acute confusional state	1 (2.8)	18 (15.5)	**0.046**
Mood disorder	1 (2.8)	8 (6.9)	0.687
Psychosis	0 (0.0)	3 (2.6)	1.000
Movement disorder	1 (2.8)	1 (0.9)	0.419
Autonomic disorder	0 (0.0)	1 (0.9)	1.000
Mononeuropathy	1 (2.8)	6 (5.2)	1.000
Cognitive dysfunction	4 (11.1)	3 (2.6)	0.055
Cranial neuropathy	0 (0.0)	4 (3.4)	0.573
Headache	3 (8.3)	7 (6.0)	0.701
Myelopathy	1 (2.8)	8 (6.9)	0.687
System involvement in SLE			
Mucocutaneous involvement	19 (52.8)	73 (62.9)	0.276
Muscle involvement	1 (2.8)	2 (1.7)	0.558
Joint involvement	9 (25.0)	52 (44.8)	**0.034**
Renal involvement	19 (52.8)	76 (65.5)	0.168
Hematological involvement	20 (55.6)	71 (61.2)	0.546
Cardiac involvement	7 (19.4)	19 (16.4)	0.670
Pulmonary involvement	5 (13.9)	12 (10.3)	0.553
Fever involvement	8 (22.2)	55 (47.4)	**0.007**
Antibodies			
Anti-Sm	4 (11.1)	21 (18.1)	0.323
Anti-dsDNA	13 (36.1)	70 (60.3)	**0.011**
Anti-SSA	13 (35.1)	72 (62.1)	**0.006**
Anti-SSB	4 (11.1)	18 (15.5)	0.512
Anti-Ro52	17 (47.2)	56 (48.3)	0.912
Anti-RiboP	4 (11.1)	34 (29.3)	**0.028**
Anti-RNP	6 (16.7)	43 (37.1)	**0.022**
Anti-Scl-70	0 (0.0)	1 (0.9)	1.000
LA	30 (83.3)	20 (17.2)	**<0.001**
aβ2GPI	34 (94.4)	3 (2.6)	**<0.001**
aCL	32 (88.9)	7 (6.0)	**<0.001**

Data for Cluster 1 and Cluster 2 are expressed as *n* (%). aCL, anticardiolipin antibody; β2GP1, β2-glycoprotein-1; dsDNA, double-stranded deoxyribonucleic acid; LA, lupus anticoagulant; NPSLE, neuropsychiatric SLE; RNP, ribonucleoprotein; RiboP, ribosomal P protein; SLE, systemic lupus erythematosus; Sm, Smith; SCL-70, topoisomerase I; SSA, Sjogren’s syndrome antigen A; SSB, Sjogren’s syndrome antigen B.

### Follow-up

**Figure 4:**
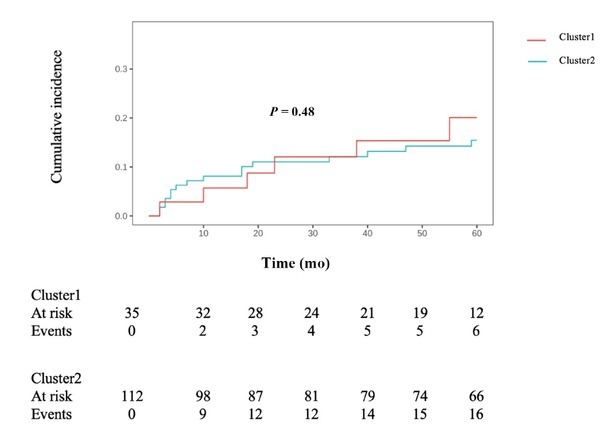
Cumulative incidence curves of NP relapse for 147 NPSLE patients. NPSLE: neuropsychiatric systemic lupus erythematosus.

Patients were followed up for a median of 60 months (IQR 22–84.5) after discharge. Long-term follow-up was conducted based on preliminary clustering results, with NPSLE recurrence as the primary endpoint (five patients were lost to follow up). In Cluster 1 (35 patients), the median follow-up duration was 63 months (IQR 22.5–63.0), 6 recurrence events (17.1%) recorded during the 5-year follow-up period. In Cluster 2 (112 patients), the median follow-up duration was 52 months (IQR 21.75–91.5), with 16 recurrence events (14.3%) observed during follow-up. Competing risks regression analysis demonstrated a trend toward a higher risk of NPSLE recurrence in Cluster 1 compared with Cluster 2 over the 5-year follow-up period, though this difference lacked statistical significance (*P* = 0.48) (Figure 4).

## Discussion

In this study, we characterized the clinical spectrum and immunological heterogeneity of primary NPSLE in a hospitalized rheumatology cohort. Using autoantibody-based hierarchical clustering, we further identified two immunologically and clinically distinct subtypes. One subtype was characterized by predominant cerebrovascular involvement and higher antiphospholipid antibody positivity. In contrast, the other subtype exhibited a systemic immune-mediated inflammatory pattern, with enrichment of anti-SSA, anti-dsDNA, and anti-RiboP antibodies and predominantly diffuse manifestations such as ACS. These findings underscore the value of immunological profiling in capturing the biological heterogeneity of NPSLE and suggest that antibody-based subtyping may contribute to improved disease classification and more informed clinical risk stratification.

NP involvement in SLE presents as a diverse and nonspecific set of symptoms. It represents a common complication and a major contributor to SLE-related morbidity and mortality.^[21,22]^ Importantly, various other factors, including infections and metabolic disorders, can also precipitate NP symptoms in patients with SLE.^[23]^ In the present study, we observed a 91% incidence of primary NPSLE among hospitalized SLE patients with NP symptoms. This higher proportion compared to previous studies ^[22,24]^ may be explained by several factors. First, hospitalization is generally reserved for patients with more severe or acute clinical presentations, and those with milder NP symptoms less likely to be admitted. Second, outpatient pre-screening helps the diversion of NP events with confirmed non-SLE etiologies—such as infections—to appropriate departments (*e.g*., infectious diseases wards). Only patients without clearly established alternative diagnoses are admitted for further evaluation.

Despite this context, determining the primary cause of NP dysfunction in SLE patients remains challenging. The ACR initially defined 19 SLE-related neuropsychiatric syndromes with exclusion and association criteria,^[8]^ which were later refined by Ainiala *et al*. for improved specificity.^[16]^ In our study, we found that 9% of hospitalized SLE patients with NP symptoms had alternative causes—most commonly infection, followed by drug toxicity and hematologic disorders. These findings are consistent with secondary/comorbid etiologies described in the Italian model.^[12]^ Regarding NP manifestations, CNS involvement predominated in our cohort (89.1%), in line with reports from global multicenter SLE cohorts.^[24, 25, 26]^ Seizure, cerebrovascular events and ACS, demyelinating syndrome constituted the main patterns of CNS involvement, whereas peripheral neuropathy was relatively uncommon, consistent with earlier reports.^[21,27, 28, 29]^ Laboratory profiling in NPSLE patients showed relatively lower serum C3 levels, elevated erythrocyte sedimentation rate (ESR), and a notable proportion with anti-SSB antibody positivity. In contrast, several other peripheral blood and CSF parameters—including IgG, CSF cell count, and protein—mostly fell within standard clinical reference ranges. These findings underscore the challenge of attributing NP symptoms to SLE in the absence of specific pathological, laboratory, or radiological markers.

Owing to its diverse clinical manifestations and diagnostic challenges, NPSLE remains dificult to characterize. By performing clustering analysis of autoantibody profiles in NPSLE patients, we explored potential immunological patterns associated with distinct clinical phenotypes, which may facilitate future diagnostic refinement.

Our findings further support the established role of APLs in NPSLE, particularly in cerebrovascular involvement. In our cohort, Cluster 1 exhibited a high prevalence of APLs and a significantly increased incidence of cerebrovascular manifestations, including both ischemic (13 events) and hemorrhagic (1 event) events. This aligns with previous reports by Giovanni *et al*. and others, which have demonstrated strong associations between APLs and obstructive or hemorrhagic cerebrovascular lesions in NPSLE.^[30, 31, 32]^ The pathogenic role of APLs in CNS involvement may involve endothelial dysfunction, thrombosis, and disruption of the blood-brain barrier, leading to vascular inflammation and ischemic injury.^[33, 34, 35]^ This mechanistic link may explain the observed clustering of vascular complications in APLs-positive individuals. It also highlights the importance of incorporating APLs testing into the routine assessment of NPSLE patients, particularly those presenting with cerebrovascular symptoms.^[36]^ Clinically, the identification of a distinct APLs-dominant subgroup (Cluster1) provides a rationale for more targeted secondary prevention strategies, including long-term anticoagulation or anti-platelet therapy in selected patients.

In contrast to the vascular-dominant profile of Cluster 1, our study identified a distinct immunologically driven subgroup (Cluster 2) characterized by significantly higher positivity of anti-SSA, anti-dsDNA and anti-RiboP antibodies. This immunological profile was accompanied by predominant NP manifestations—particularly ACS and a higher frequency of systemic symptoms such as fever and arthritis. These findings suggest that Cluster 2 may represent a more inflammatory phenotype of NPSLE, with heightened autoimmune activity across multiple pathways. Previous studies have implicated anti-RiboP and anti-dsDNA antibodies in the pathogenesis of CNS involvement in SLE, particularly in relation to mood disorders and cognitive dysfunction,^[37, 38, 39, 40, 41, 42]^ this finding was further supported by a cluster analysis of a Turkish juvenile NPSLE cohort.^[43]^ The association between these antibodies and ACS in Cluster 2 suggests a potential link to neuroinflammatory processes. Anti-SSA antibodies, while traditionally associated with cutaneous and sicca symptoms,^[44,45]^ have also been linked to more systemic immune dysregulation and may contribute to broader inflammatory cascadesin NPSLE.^[46, 47, 48]^

These subtype-specific immunological and clinical features may provide important clues for therapeutic decision-making, particularly given the diagnostic complexity and limited disease-specific evidence in NPSLE. Once primary NPSLE is established, treatment strategies may be guided by the predominant pathogenic processes, distinguishing an ischemic/ APLs-dominant subtype from an inflammatory/autoantibody-dominant subtype. For the ischemic/APLs-dominant subtype, antithrombotic approaches are commonly considered in clinical practice, especially in patients with persistent aPL positivity. In our cohort, this subtype was associated with a recurrence rate of 17.1%, suggesting that sustained secondary prevention may be warranted in selected high-risk patients.^[49]^ In contrast, the inflammatory/autoantibody-dominant subtype appears to benefit more from immunosuppressive therapy. High-dose glucocorticoids combined with CTX is preferred for severe cases, with prior studies reporting favorable outcomes compared with glucocorticoids alone,^[50]^ oral CTX followed by azathioprine is effective for lupus psychosis.^[51]^ Rituximab is a consideration for refractory disease, supported by evidence from adult ^[52]^ and pediatric ^[53]^ cohorts, while belimumab lacks evaluation in major CNS manifestations.^[29]^ Notably, high-level evidence for NPSLE treatment remains limited,^[54]^ underscoring the need for future research to conduct more randomized controlled trials (RCTs) comparing immunosuppressive regimens for NPSLE and explore additional novel therapeutic strategies.

The competing risks regression model revealed that during the 60-month follow-up period, Cluster1 exhibited a 36% increased risk of NPSLE recurrence compared to Cluster2 (*P* = 0.48); however, this difference did not reach statistical significance. To date, no published studies have explicitly demonstrated a higher recurrence risk in the cerebrovascular disease subtype than in other NPSLE subtypes. The observed trend in our cohort may therefore be influenced by the limited sample size. Future studies with expanded cohorts will be necessary to validate these findings more robustly.

Although the pathogenesis of NPSLE remains complex and incompletely understood, current evidence supports two predominant pathways: ischemic injury and autoimmune-mediated neuroinflammatory cascades.^[55]^ In our clustered cohorts, clinical features in Cluster 2 were more consistent with neuroinflammatory processes, while Cluster 1 primarily reflected ischemic mechanisms—paralleling established pathogenic frameworks for NPSLE. Together, these findings further highlight the biological heterogeneity of NPSLE and underscore the potential utility of immunological phenotyping in informing future diagnostic refinement and therapeutic strategies.

We also acknowledge several limitations of this study. First, the relatively small sample size and single-center design may limit the statistical power and generalizability of the findings. In addition, the cohort consisted predominantly of hospitalized patients, excluding milder outpatient cases, which may introduce selection bias. Second, the retrospective design may have affected the completeness and consistency of data collection. To strengthen the robustness of these findings, further large-scale, multicenter studies are warranted. Despite these limitations, our study provides valuable insights into the clinical and immunological heterogeneity of NPSLE. Importantly, the application of clustering analysis enabled the stratification of patients into distinct subgroups, facilitating the simplification of complex datasets and the identification of meaningful patterns across laboratory findings, clinical manifestations, and disease outcomes. This approach may inform future efforts toward personalized risk assessment and the development of more targeted therapeutic strategies for patients with NPSLE.

## Conclusions

NPSLE demonstrated distinct serological features and segregated into two immunologically defined clusters in this study. These findings underscore the clinical and biological heterogeneity of NPSLE and suggest that immunological profiling may enhance precise classification and personalized management of affected patients.

## Supplementary Material

Supplementary Material Details
