# Supplementary material for: Neuropsychiatric systemic lupus erythematosus subtypes identified by unsupervised clustering: A single-center cohort study
Source: Rheumatol Immunol Res. 2026 Apr 8;7(1):14–23. doi: 10.1515/rir-2026-0002 (PMC13066665; doi:10.1515/rir-2026-0002)
Supplement: Supplementary file 1 — Supplementary Material Details [file rir-2026-0002_sm.pdf]

Supplementary material

Supplementary Table S1. Distribution of NP manifestations

**Type of NP manifestations**	**NPSLE patients (*n* = 152)**
Isolated CNS, *n* (%)	127 (83.5)
Multiple CNS, *n* (%)	22 (14.5)
Single CNS, *n* (%)	105 (69.0)
Isolated PNS, *n* (%)	17 (11.2)
Multiple PNS, *n* (%)	1 (0.7)
single PNS, *n* (%)	16 (10.5)
Combined CNS and PNS, *n* (%)	8 (5.3)

CNS: central nervous system; PNS: peripheral nervous system; NP: neuropsychiatric; NPSLE: neuropsychiatric systemic lupus erythematosus.

Supplementary Table S2. Attributing all NP events to SLE

**NPSLE subtypes**	**NPSLE events (*n* =187)**
**CNS**	161(89.1%)
Aseptic meningitis	11(5.9%)
Cerebrovascular disease	36(19.3%)
Demyelinating syndrome	15(8.0%)
Headache	10(5.3%)
Movement disorder	2(1.1%)
Myelopathy	9(4.8%)
Seizure disorder	39(20.9%)

Acute confusional state	19(10.2%)
Anxiety	1(0.5%)
Cognitive dysfunction	7(3.7%)
Mood disorder	9(4.8%)
Psychosis	3(1.6%)
**PNS**	26 (13.9%)
Guillain–Barré syndrome	0(0.0%)
Autonomic disorder	1(0.5%)
Mononeuropathy	7(3.7%)
Myasthenia gravis	0(0.0%)
Cranial neuropathy	4(2.1%)
Plexopathy	0(0.0%)
Polyneuropathy	14(7.5%)

CNS: central nervous system; PNS: peripheral nervous system.

Supplementary Table S3. Basic characteristics of patients undergoing lumbar puncture

**Characteristic**	**NPSLE underwent LP (*n* = 139)**
Pressure of CSF (mmH_2O, median [IQR])	155 [120–195]
Increased ICP, n (%)	46 (33.1)
WBC of CSF ($\times 10^6$/L, median [IQR])	2 [0–3]
Protein of CSF (g/L, median [IQR])	0.39 [0.28–0.57]
Increased CSF protein, *n* (%)	53 (38.1)
Cl (mmol/L, median [IQR])	124 [122–126]
Glu (mmol/L, median [IQR])	3.1 [2.80–3.70]

CSF: cerebrospinal fluid; ICP: Intracranial pressure.

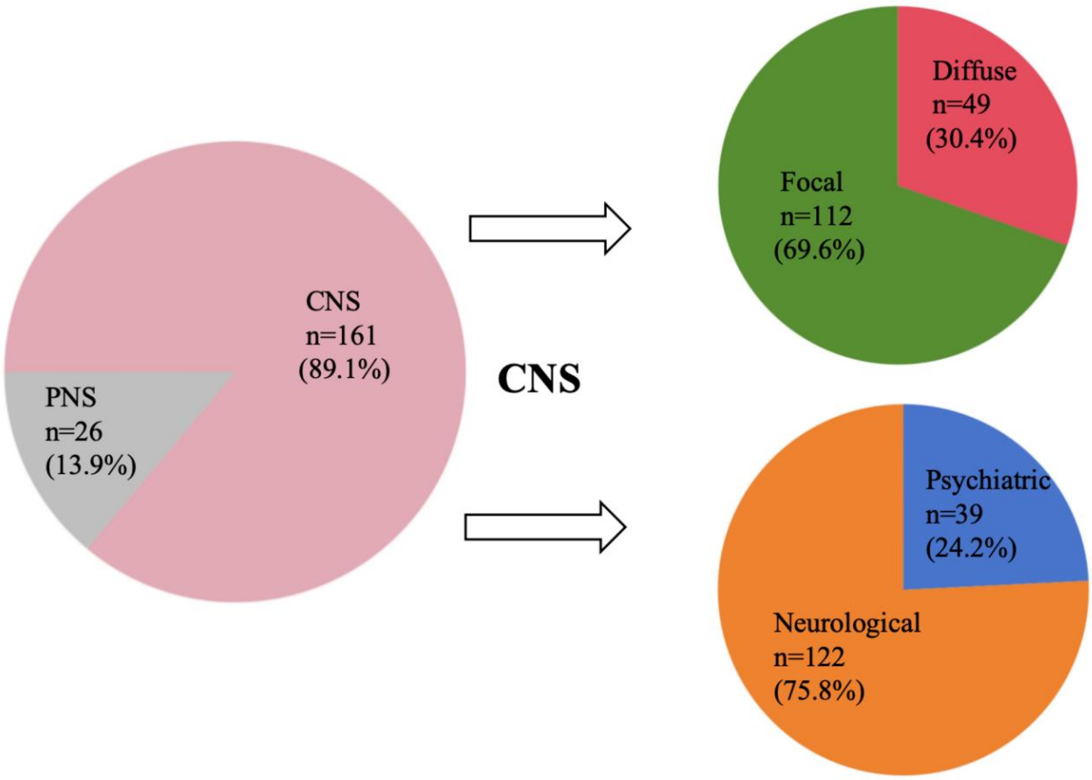

Supplementary Figure S1. Distribution of CNS and PNS Manifestations in NPSLE (with CNS Subtypes Shown as Pie Percentages). CNS: central nervous system; PNS: peripheral nervous system

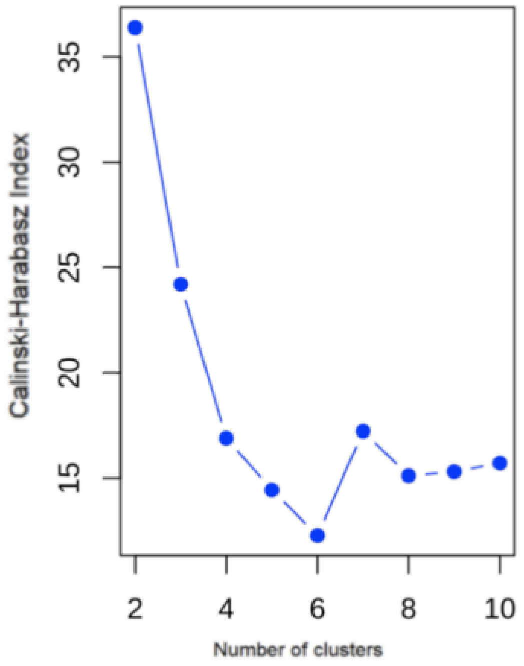

Supplementary Figure S2. Calinski–Harabasz index for determining the optimal cluster number.
